# Autonomous Inhibition of Apoptosis Correlates with Responsiveness of Colon Carcinoma Cell Lines to Ciglitazone

**DOI:** 10.1371/journal.pone.0114158

**Published:** 2014-12-11

**Authors:** David M. Baron, Ulrike Kaindl, Verena J. Haudek-Prinz, Editha Bayer, Clemens Röhrl, Christopher Gerner, Brigitte Marian

**Affiliations:** 1 Department of Medicine I, Clinic for Internal Medicine I, Institute of Cancer Research, Medical University of Vienna, Vienna, Austria; 2 Department of Anesthesia, General Intensive Care, and Pain Management Medical, Medical University of Vienna, Vienna, Austria; 3 Institute of Analytical Chemistry, Faculty of Chemistry, University of Vienna, Vienna, Austria; University of Kansas Medical Center, United States of America

## Abstract

Colorectal cancer is a leading cause of mortality worldwide. Resistance to therapy is common and often results in patients succumbing to the disease. The mechanisms of resistance are poorly understood. Cells basically have two possibilities to survive a treatment with potentially apoptosis-inducing substances. They can make use of their existing proteins to counteract the induced reactions or quickly upregulate protective factors to evade the apoptotic signal. To identify protein patterns involved in resistance to apoptosis, we studied two colorectal adenocarcinoma cell lines with different growth responses to low-molar concentrations of the thiazolidinedione Ciglitazone: HT29 cells underwent apoptosis, whereas SW480 cells increased cell number. Fluorescence detection and autoradiography scans of 2D-PAGE gels were performed in both cell lines to assess protein synthesis and turnover, respectively. To verify the data we performed shotgun analysis using the same treatment procedure as in 2D-experiments. Biological functions of the identified proteins were mainly associated with apoptosis regulation, chaperoning, intrinsic inflammation, and DNA repair. The present study suggests that different growth response of two colorectal carcinoma cell lines after treatment with Ciglitazone results from cell-specific protein synthesis and differences in protein regulation.

## Introduction

Behind infectious and cardiovascular diseases, cancer is the third leading cause of mortality worldwide, accounting for 7.6 million (13%) of all deaths. Colorectal cancer (CRC) is the third most frequently diagnosed malignant disease with over 1 million new cases and more than 600,000 deaths each year [1]. Despite new chemotherapeutic regimens disease-specific mortality from CRC remains high [2], [3], validating intensified research in this field. As a consequence, numerous substances are being investigated for possible anti-cancerous effects.

One of these substance groups are thiazolidinediones (TZDs), agonists of the peroxisome proliferator-activated receptor (PPAR)-γ. The first synthesized member, Ciglitazone (CIG), showed insulin-sensitizing effects [4], and several derivatives have been approved for treatment of non-insulin-dependent diabetes mellitus [5]. Peroxisome proliferator-activated receptors are involved in the regulation of lipid metabolism and reactions related to energy homeostasis [6], as well as wound repair and inflammation [7], [8]. These actions imply involvement in cell proliferation and differentiation, making PPARs interesting targets for cancer treatment. Especially PPAR-γ, which mediates differentiation of fibroblast [9] and muscle cells [10] to adipocytes after retroviral transfection, has been investigated for therapeutic interventions [11].

Activation of the PPAR-γ gene in models of CRC has produced varying results. Both reduction of tumor growth and size [12]–[14] and a survival effect in tumor cells have been described [15], [16]. These different survival responses after treatment with PPAR-γ agonists might be caused by deregulation of apoptosis at the mitochondrial level or during downstream conduction of apoptotic signals. Several proteins can regulate both survival and apoptosis during phases with increased cellular stress [17]. However, complex interactions between pro- and anti-apoptotic proteins make it difficult to elucidate the exact pathways involved in the cell's response to therapeutic agents.

In recent years, there has been a trend to make use of broad screening techniques, enabling scientists to survey whole genomes and proteomes. Several attempts have already been ventured to profile human cancer cell lines using microarray systems [18], [19] or proteomic approaches [20]–[25]. Proteomic studies, however, set their focus on total protein amounts, which are not always adequate to characterize cell-reactions after stimulation. As previously described, we established a technique to profile dynamic changes in the proteome [26], [27] using metabolic labeling with ^35^S-methionine. This method can e.g. quantify protein synthesis rates of different cell lines after drug treatment.

In the current study, we examined the poorly differentiated human cell lines HT29 and SW480, both derived from colorectal adenocarcinomas. These cell lines originate from two different patients, and show varying growth rates and shape characteristics. The SW480 cell line has a shorter replication time (20–24 hours as compared to 36–40 hours in HT29 cells) and a higher invasive potential [28]. We report diverse responsiveness of HT29 and SW480 cells after treatment with low-molar concentrations of TZDs. In order to elucidate potential mechanisms responsible for this differing responsiveness, we performed 2D-PAGE of untreated cells. Additionally, we determined protein synthesis rates after treatment of cell lines with CIG using labeling with 35S-methionine. To further confirm the existence of the identified proteins we performed shotgun analysis of both untreated and treated CRC cell cultures.

## Materials and Methods

### Tissue culture

The colon adenocarcinoma cell lines HT29 (ATCC HTB-38) and SW480 (ATCC CCL-228) were obtained directly from the American Tissue Culture Collection (Rockville, MD) and routinely cultivated under standard tissue culture conditions (humidified atmosphere of 95% air +5% CO_2_ at 37°C) using minimal essential medium (MEM) containing 10% fetal bovine serum, 100 U/ml penicillin, and 100 mg/ml streptomycin (PAA, Pasching, Austria). For experiments, the medium was replaced with serum-free MEM containing either vehicle control (DMSO) or indicated concentrations of the PPAR-γ agonists Ciglitazone (CIG), Troglitazone (TRO), and Rosiglitazone (ROSI) or the PPAR-γ antagonist GW9662 (all from Cayman Chemical, Ann Arbor, MI). The natural PPAR-γ agonists Prostaglandin J_2_ (PGJ_2_) was purchased from Sigma (St. Louis, MO) and used as a positive control. All cell culture experiments were performed independently three times from different passages.

### Luciferase assay

Cells were transfected with pTransLucent/PPAR gamma reporter vector and pTransLucent control vector expressing renilla luciferase under the control of a CMV promoter (Panomics, Redwood City, CA). After 24 hours cells lysates were obtained using Passive Lysis Buffer provided with the Dual Luciferase assay (Promega Madison, WI) and luciferase activity determined according to the manufacturer's instructions. In short: 20 µl of the cleared supernatants were transferred to a white microplate for luminescence (Greiner, Bio-One, Frickenhausen, Germany) and mixed with 100 µl Luciferase Assay Reagent II. Firefly luciferase was measured for ten seconds in a luminometer (BioTek Instruments, Winooski, VE). The same measurement was performed for Renilla luciferase after adding 100 µl Stop & Glo Reagent.

### Cell viability assay

Cells were treated with TZDs for 6 or 24 hours. Cell number was determined by neutral red (Merck, Darmstadt, GER) uptake over 2 hours in serum-free MEM containing 50 µg/ml of the dye, which diffuses into the lysosomes of viable cells. After removal of excess dye with phosphate-buffered saline, amounts proportional to the number of viable cells can be extracted with 1% acetic acid in 70% ethanol. Extinction was measured photometrically in 96-well-plates at 562 nm with 620 nm as reference.

### Assessment of apoptosis and necrosis

Cells were washed with PBS/EDTA and detached from the plates by trypsinisation. Resuspended cells were incubated with 10 µg/ml JC-1 (dissolved in 10%-FCS-MEM) at 37°C for 10 minutes. A loss of mitochondrial membrane potential (Δψ_m_) can be observed in cells entering apoptosis. This reaction results in a shift of JC-1 fluorescence, which was analyzed by FACS.

Necrosis was assessed using the lactate dehydrogenase (LDH) release assay (Roche Diagnostics, Mannheim, Germany). Triton X-100-treated cultures were used as a positive control.

### Cell cycle distribution

To isolate nuclei, cells were washed with PBS/EDTA, detached by trypsinisation and homogenized in cold nuclear isolation buffer (0.5 M acetic acid, 0.5% Tween 20). Nuclei were collected by centrifugation for 5 minutes at 650 g and 4°C and then resupended in PBS containing 0.1 mg/ml RNAse and 50 µg/ml propidium iodide. DNA content of isolated nuclei was analyzed by flow cytometry using a CALIBUR flow cytometer (Becton Dickinson, Sunnyvale, CA) equipped with 15 mW 488 nm and 633 nm argon lasers. Data were analyzed with CELLQUEST and MOD-FIT software (Beckton Dickinson).

### Sample preparation and sub-cellular fractionation for 2D-PAGE

Tissue cultures were treated with 5 µM CIG for 6 hours. Subsequently, cells were incubated at 37°C in methionine- and cysteine-free RPMI-1640 containing CIG 5 µM supplemented with 0.2 mCi/ml ^35^S-labelled methionine and cysteine (Trans35Slabel, Biomedica, MP Biomedicals) for 2 hours. For the isolation of cytoplasmic proteins, all buffers were supplemented with protease inhibitors PMSF (1 mM), aprotinin, leupeptin, and pepstatin A (each at 1 µg/ml). Cells were lysed in lysis buffer (10 mM HEPES/NaOH, pH 7.4, 0.25 M sucrose, 10 mM NaCl, 3 mM MgCl_2_, 0,5% Triton X-100). The cytoplasmic fraction was separated from the nuclei by centrifugation at 2.000 g for 5 minutes and ethanol precipitated. Protein samples were dissolved in sample buffer (7.5 M urea, 1.5 M thiourea, 4% CHAPS, 0.05% SDS, 100 mM DTT).

### 2D-PAGE

Proteins were loaded by passive rehydration of IPG strips pH 5–8, 17 cm (Bio-Rad, Hercules, CA) at room temperature. IEF was performed in a stepwise fashion (1 h 0-500 V linear; 5 h 500 V; 5 h 500–3500 V linear; 12 h 3500 V). After IEF, the strips were equilibrated with 100 mM DTT and 2.5% iodacetamide according to the instructions of the manufacturer (Bio-Rad). For SDS-PAGE using the Protean II xi electrophoresis system (Bio-Rad), the IPG strips were placed on top of 1.5 mm 12% polyacrylamide slab gels and overlaid with 0.5% low melting agarose. The gels were stained with a 400 nM solution of Ruthenium II tris (bathophenanthroline disulfonate; RuBPS) as described by Rabilloud et al. [29]. Fluorescence detection scanning was performed with the FluorImager 595 (GE Healthcare, Fairfield, CT) at a resolution of 100 µm using the recommended settings for SYPRO Ruby detection. After scanning, gels were dried using the slab gel dryer SE110 (Hoefer, San Francisco, CA). Exposure of storage phosphor screens (Molecular Dynamics, Sunnyvale, CA) occurred at room temperature for 24 hours. Screens were subsequently scanned using the Phosphorimager SI (Molecular Dynamics) at a resolution of 100 µm.

Gels were aligned using TT900-S2S warping software (version 2006; Nonlinear Dynamics, Newcastle upon Tyne, UK). Quantitative analysis was performed with Progenesis PG-200 (Nonlinear Dynamics) using SameSpots technology. Three different gels derived from independent experiments were chosen for statistical analysis, which was done with students t-test included in the software package. All 2D-gel data were independently reproduced for at least three times.

### 1D-PAGE for shotgun analysis

The cytoplasmic protein fraction was loaded on the slots of a 12% polyacrylamide gel, electrophoresis was carried out until complete separation of the molecular marker (Dual Color, Bio-Rad). Gels were fixed with 50% methanol/10% acidic acid and subsequently silver stained as described below. The different lanes were cut out of the gel with respect to the molecular weights shown by the molecular marker and the respective gel-pieces were digested with trypsin.

### Silver-staining

1D-gels were fixed with 50% methanol, washed and sensitized with 0.02% Na_2_S_2_O_3_. Gels were then stained with ice-cold 0.1% AgNO_3_ for 20 minutes, rinsed with bidestillated water and subsequently developed with 3% Na_2_CO_3_/0.05% formaldehyde as previously described [30].

### Tryptic digestion

Protein spots were cut out of the gel, the gel-pieces were stripped with 15 mM K_3_Fe(CN)_6_/50 mM Na_2_S_2_O_3_ and intensively washed with 50% methanol/10% acetic acid. The pH was adjusted with 50 mM NH_4_HCO_3_. In case of shotgun samples, the proteins were reduced with 10 mM DTT/50 mM NH_4_HCO_3_ for 30 minutes at 56°C and finally alkylated with 50 mM iodacetamide/50 mM NH_4_HCO_3_ 20 minutes in the dark. Afterwards the gel-pieces were dried with acetonitrile and accelerated dried in the speedvac. Between each step, the tubes were shaken 5–10 minutes (Eppendorf Thermomixer comfort). The dried gel-spots were treated with trypsin 0.1 mg/ml (Trypsin sequencing grade, Roche Diagnostics)/50 mM NH_4_HCO_3_, in a ration of 1∶8 for 20 minutes on ice, covered with 50 mM NH_4_HCO_3_ and were subsequently stored over night at 37°C. The digested peptides were eluted by adding 50 mM NH_4_HCO_3_ to the gel-spots, the supernatant transferred into silicon-coated tubes, and the extraction procedure repeated for two times with 5% formic acid/50% acetonitril. Between each elution step the gel-spots were sonicated. Finally the peptide solution was concentrated to an appropriate volume [31].

### Protein identification

Mass spectrometry was performed as described previously [32]. In short, peptides were separated by nano-flow LC (1100 Series LC system, Agilent, Palo Alto, CA) using the HPLC-Chip cube (Agilent) equipped with a 40 nl Zorbax 300SB-C18 trapping column and a 75 µm×150 mm Zorbax 300SB-C18 separation column at a flow rate of 400 nl/minute, using a gradient from 0.2% formic acid and 2% ACN to 0.2% formic acid and 50% ACN over 80 minutes. Peptide identification was accomplished by MS/MS fragmentation analysis with an iontrap mass spectrometer (XCT-Ultra, Agilent) equipped with an orthogonal nanospray ion source. The MS/MS data, including peak list-generation and search engine, were interpreted by the Spectrum Mill MS Proteomics Workbench software (Version A.03.03, Agilent) allowing for two missed cleavages and searched against the SwissProt Database for human proteins (Version 12/2010 containing 20328 entries) allowing for precursor mass deviation of 1.5 Da, a product mass tolerance of 0.7 Da and a minimum matched peak intensity of 70%. Due to previous chemical modification, carbamidomethylation of cysteine was set as fixed modification; methionine oxidation was allowed as variable modification. Data interpretation was supported by Griss Proteomics database Engine [33]. Peptides with SpectrumMill scores greater than 13 were included in the result files. Peptides scoring between 9 and 13 were included if precursor m/z value, retention time, and MS2 pattern matched to a reference spectrum scoring above 13.

### Statistical analysis

All experiments were performed at least in triplicate. The results are reported as mean values ± standard deviation. Statistical analysis was performed using GraphPad Prism 6 software (GraphPad Software, La Jolla, CA). Statistical differences were determined using two-tailed student's t-test or ANOVA, as indicated. P-values <0.05 were considered significant.

## Results and Discussion

### Differences in cell viability and induction of apoptosis between colorectal cancer cell lines in response to treatment with PPAR-γ agonists

To assess the efficiency of PPAR-γ activation in colorectal tumor cells, HT29 and SW480 colon carcinoma cell lines were exposed to CIG, TRO, and ROSI, as well as PGJ2. The promoter activity of PPRE was assessed by dual luciferase assay using a PPRE-reporter construct. In HT29 cells, incubation with 5 µM of TZDs doubled reporter gene expression (S1A Figure). At higher concentrations, activity did not increase further (CIG and ROSI) or even decreased (TRO). The positive control PGJ_2_ caused a 17-fold induction of the PPRE reporter. In SW480 cells, all three TZDs induced reporter gene expression in a concentration-dependent manner (S1B Figure). The positive control PGJ_2_ caused a 28-fold induction of the PPRE reporter. Activation of reporter gene expression could be partially blocked by pre-treatment of the cultures with the PPAR-γ antagonist GW9662 for 3 hours at a concentration of 10 µM (S1C Figure).

**Figure 1:**
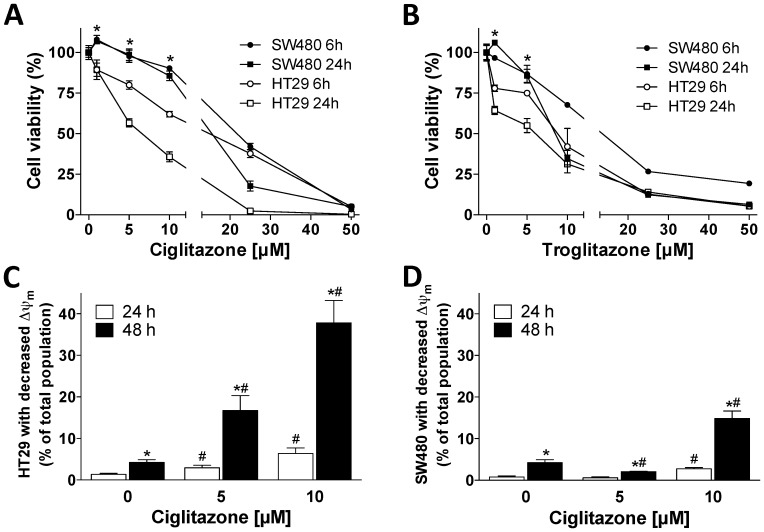
Cell viability and mitochondrial membrane potential after treatment with thiazolidinediones. Cell viability was assessed by neutral red uptake in SW480 and HT29 cells treated with increasing concentrations of (A) Ciglitazone or (B) Troglitazone for 6 and 24 hours. *p<0.05, values of HT29 cells differ from those of SW480 cells. Mitochondrial membrane potential (Δψ_m_) was assessed by JC-1 FACS analysis in (C) HT29 and (D) SW480 cells treated with increasing concentrations of Ciglitazone for 24 and 48 hours. *p<0.05, values at 48 hours differ from those at 24 hours. #p<0.05, values at indicated concentrations differ from baseline.

Several research groups have reported apoptosis- and differentiation-inducing effects of PPAR-γ activators in colorectal cancer cell lines [34], [35]. To confirm these data we performed neutral red viability assays after incubation of HT29 and SW480 cells with TZDs. In HT29 cells, CIG induced a dose-dependent decrease of cell number after 6 and 24 hours (Fig. 1A). Surprisingly, in SW480 cells concentrations of CIG less than 5 µM, which is around the published EC_50_ for CIG binding to the PPAR-γ, caused a small but highly reproducible increase in cell number. These effects of CIG were potentiated even further after 48 and 72 hours in both cell lines (data not shown). The effects of TRO on cell number were similar. Incubation of HT29 cells with TRO caused a dose-dependent decrease of cell number after 6 and 24 hours (Fig. 1B). In SW480 cells, cell numbers were consistently increased by about 10% at 0.5 µM, which is the EC_50_ for TRO.

To determine whether differences in cell number between HT29 and SW480 cells at low-molar concentrations of CIG could be explained by different apoptotic responses, Δψ_m_ was determined by FACS analysis using JC-1 after 24 and 48 hours of incubation with CIG. In HT29 cells, treatment with CIG increased the percentage of cells with reduced Δψ_m_ in a time- and concentration-dependent manner (Fig. 1C). Contrarily, in SW480 cells incubation with 5 µM CIG for 24 hours did not change the percentage of cells with reduced Δψ_m_ (1±0% vs. 1±0%, Fig. 1D). Incubation with 5 µM CIG for 48 hours even reduced the percentage of SW480 cells with reduced Δψ_m_ (4±1% vs. 2±1%, p<0.05). At concentrations of 10 µM CIG, the percentage of cells with reduced Δψ_m_ increased in SW480 cells, albeit to a lesser extent than HT29 cells. These results are in agreement with those obtained in the viability assays. Thus, the different responsiveness of HT29 and SW480 cells to treatment with low-molar concentrations of CIG is at least in part mediated by varying apoptotic regulation.

In order to investigate whether cell death occurred via necrosis after treatment with CIG, the LDH release into the medium was measured. The LDH release after 24 hours was <10% of the maximum activity in both untreated and treated cells, and did not differ between controls and cells treated with CIG (S2 Figure). These results indicate that cell death via necrosis is negligible after treatment of HT29 and SW480 cells with CIG, even at concentrations as high as 20 µM.

### Differences in baseline protein synthesis between HT29 and SW480 cell lines

**Figure 2:**
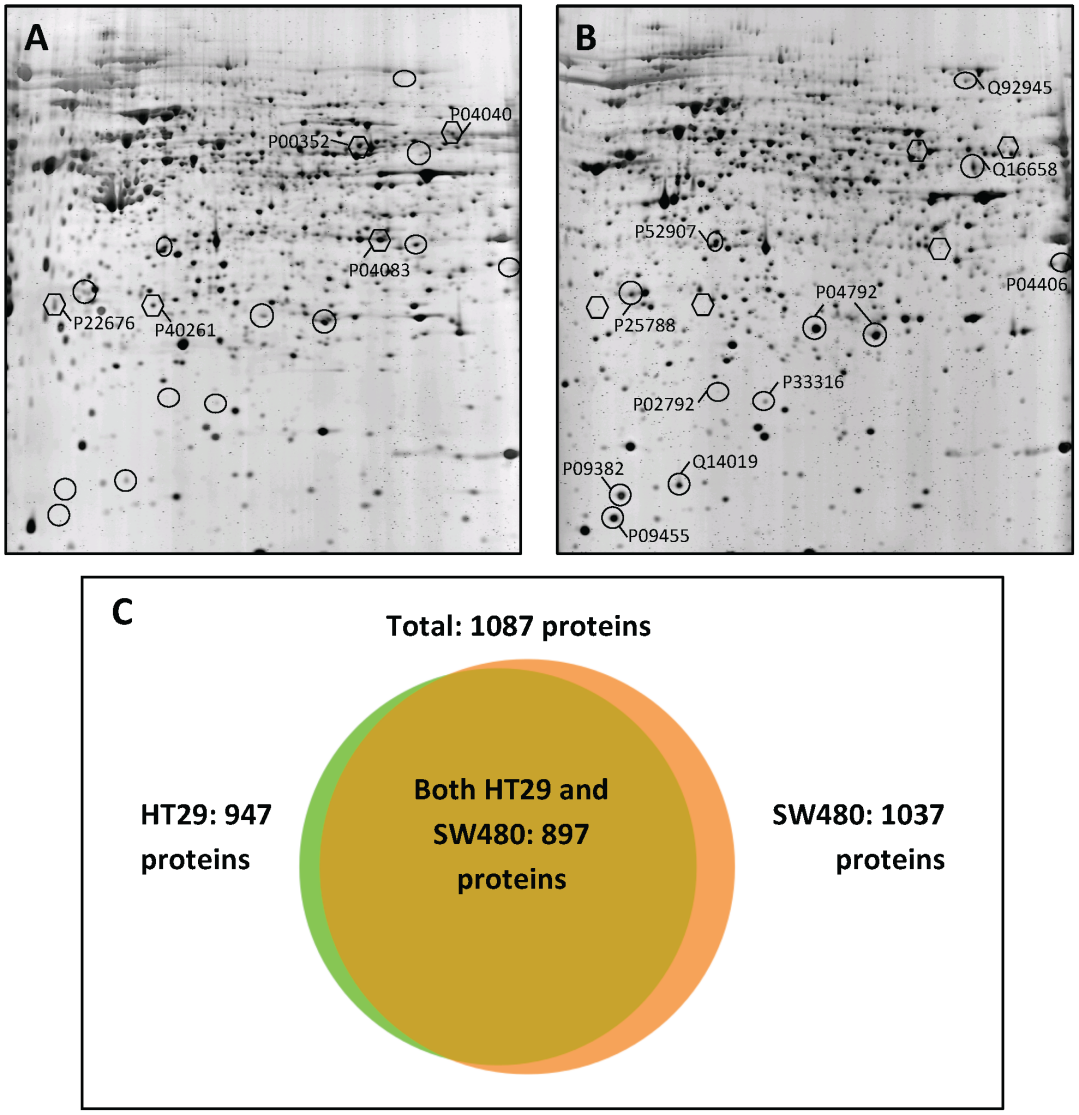
Protein synthesis in untreated colorectal adenocarcinoma cell lines. Representative 2D-PAGE gels of untreated (A) HT29 and (B) SW480 cells. Proteins synthesized to a greater extent in HT29 cells are indicated by hexagons, those in SW480 cells by circles. Accession numbers of proteins listed in Table 1 are annotated according to the UniProtKB/Swiss-Prot database. (C) Total proteins identified by shotgun analysis in HT29 and SW480 cells.

Therefore, our first goal was to compare protein synthesis patterns between untreated cell lines in order to recognize proteins, which are constantly expressed by the cells and might explain survival-promoting effects. In order to elucidate whether baseline alterations in protein synthesis might be associated with the different responsiveness to treatment with CIG, 2D-PAGE with fluorescence detection was performed in untreated HT29 and SW480 cell lines. Overall, 5 cytoplasmic proteins were synthesized at least three-fold higher in HT29 cells than in SW480 cells (p<0.05, Table 1 and Fig. 2A). In comparison, 12 cytoplasmic proteins were synthetized at least three-fold higher in SW480 cells than in HT29 cells (p<0.05, Table 1 and Fig. 2B), whereas 570 proteins were identified in both cell lines with less than three-fold difference. Gel-to-gel variations, however, pose an important limitation for proteomic research. When tissue culture, cell preparation, and 2D-gel-runs are replicated independently slight variations cannot be avoided [36].

**Table 1:** Proteins differently synthesized between HT29 and SW480 cells as determined by 2D-PAGE fluorescence.

Proteins with greater synthesis in HT29 cells	Acc.No.	Diff.	p-value	SG
Annexin A1	P04083	21,11	0.001	✓
Retinal dehydrogenase 1	P00352	7,56	0.014	✓
Nicotinamide N-methyltransferase	P40261	3,77	0.004	✓
Calretinin	P22676	3,51	0.027	✓
Catalase	P04040	3,21	0.020	✓

Proteins with greater synthesis in SW480 cells	Acc.No.	Diff.	p-value	SG
Retinol-binding protein 1, cellular	P09455	56,37	0,017	✓
Galectin-1	P09382	26,11	0,002	✓
Deoxyuridine 5'-triphosphate nucleotidohydrolase	P33316	6,43	0,002	
Proteasome subunit alpha type 3	P25788	5,47	0,001	
Fascin	Q16658	4,3	0,001	✓
Heat-shock protein beta-1 (HSP 27), isoform 2	P04792	4,04	0,012	✓
F-actin capping protein alpha-1 subunit	P52907	3,88	0,024	
Heat-shock protein beta-1 (HSP 27), isoform 1	P04792	3,71	0,002	✓
Glyceraldehyde-3-phosphate dehydrogenase	P04406	3,71	0,019	✓
Far upstream element binding protein	Q92945	3,42	0,007	
Ferritin light chain	P02792	3,26	0,012	✓
Coactosin-like protein	Q14019	3,06	0,003	✓

Checkmarks indicate correlation with shotgun analysis (SG).

Thus, the same protein samples were analyzed using a shotgun approach. Shotgun experiments pose a good way to validate 2D-data of total protein synthesis. All 12 proteins listed in Table 1 were also detected with shotgun analysis. Eight of the 12 proteins identified as differently synthesized between the two cell lines in 2D-PAGE were confirmed by 1D-shotgun analysis (Table 1). The other four proteins could also be identified by mass spectrometry, but showed no significant difference in total baseline amount between HT29 and SW480 cells. These observed differences in 2D-gels may result from a varying extent of modifications such as phosphorylation. Most proteins were identified in both cell lines (Fig. 2C and S1 Table). Additionally, 50 proteins were reproducibly identified in HT29 cells and 140 proteins in SW480 cells only. Remarkably, three cytoplasmic proteins specifically identified in SW480 cells are well-known pro-apoptotic factors: caspase-3, APAF-1 and apoptosis-inducing factor 1 (S2 Table). This observation suggests that apoptotic pathways in SW480 cells have become deregulated. It appears as though intrinsic pro-apoptotic signals are active, but fail to generate downstream responses, as previously reported in lung cancer and intestinal epithelial cells [37], [38]. Since cell signaling in cancer cells is deregulated, these pro-apoptotic signals might be blocked downstream of caspase activation by inhibitor of apoptosis signaling [39]. Thus, rather weak pro-apoptotic signals, such as those induced by treatment with 5 µM CIG in HT29 cells, might not be enough to trigger apoptosis in SW480 cells.

Another mechanism allowing cells to resist death-inducing stimuli is upregulation of chaperones [40]. Both HT29 and SW480 cells are adenocarcinomas of the colon, and show high synthesis of heat shock proteins [41]. However, two isoforms of HSP27 were synthetized to a greater extent by SW480 cells at baseline. Recently, Yang et al. reported that resistance of a human gastric carcinoma cell line to vincristine is associated with higher synthesis of HSP27, and that suppression of HSP27 enhances chemosensitivity to this drug [42]. Furthermore, Cocannon et al. have demonstrated that HSP27 inhibits cytochrome c-mediated caspase activation by sequestering pro-caspase-3 and cytochrome c [43]. Thus, the increased synthesis of HSP27 in SW480 cells might be in part responsible for the survival response of these cells after treatment with low-molar concentrations of CIG.

A recent review by Latz et al. has highlighted the similarities between the apoptosome and the inflammasome [44]. Prolonged exposure of cells to inflammation and its mediators might result in adaptation to such stressors by phenotypic alterations, thereby possibly enhancing cellular survival. Mahalingaiah et al. recently reported that chronic oxidative stress increases tumorigenic and metastatic potential in breast cancer cells, whereas acute exposure to reactive oxygen species inhibited cell growth in a dose-dependent manner [45]. In addition, studies of proliferative and malignant diseases have shown that intracellular reactive oxygen species induce an antioxidant gene expression via upregulation of anti-apoptotic proteins [46], [47]. Several proteins exclusively found in SW480 cells suggest the presence of a constant intrinsic inflammation signature in these cells. One of these proteins is Casein kinase II, whose expression is enhanced in animal models of chronic colitis [48]. Enhanced casein kinase II activity promotes epithelial restitution and protects intestinal epithelial cells from cytokine-induced apoptosis. Furthermore, upregulation of ICAM-1 [49] and S100 A13 [50] has been associated with a more aggressive phenotype in CRC tissues. In addition, the double-strand DNA repair protein RAD23 [51], the inflammation-associated DNA repair protein MSH6 [52], and the replication factor MCM4 [53] were only identified in SW480 cells (S2 Table). These proteins are involved in DNA repair, indicating the increased potential of SW480 cells to deal with stressors resulting in DNA damage.

Another protein synthesized by SW480 but not HT29 cells is Galectin-1. Previous publications indicate that Galectin-1 is not only linked to the organization of cell structure, but that overexpression of Galectin-1 is associated with the neoplastic progression in CRC [54]. Paz et al. have shown that Galectin-1 anchors ras to the plasma membrane and thereby enhances cell transformation [55]. In SW480 cells ras is constitutively activated, whereas in HT29 cells wild-type ras is expressed [56]. This constitutive activation of ras in SW480 cells can thus activate tumor-promoting signals and further enhance tumor progression.

### Protein regulation and cell cycle distribution after treatment with Ciglitazone

**Figure 3:**
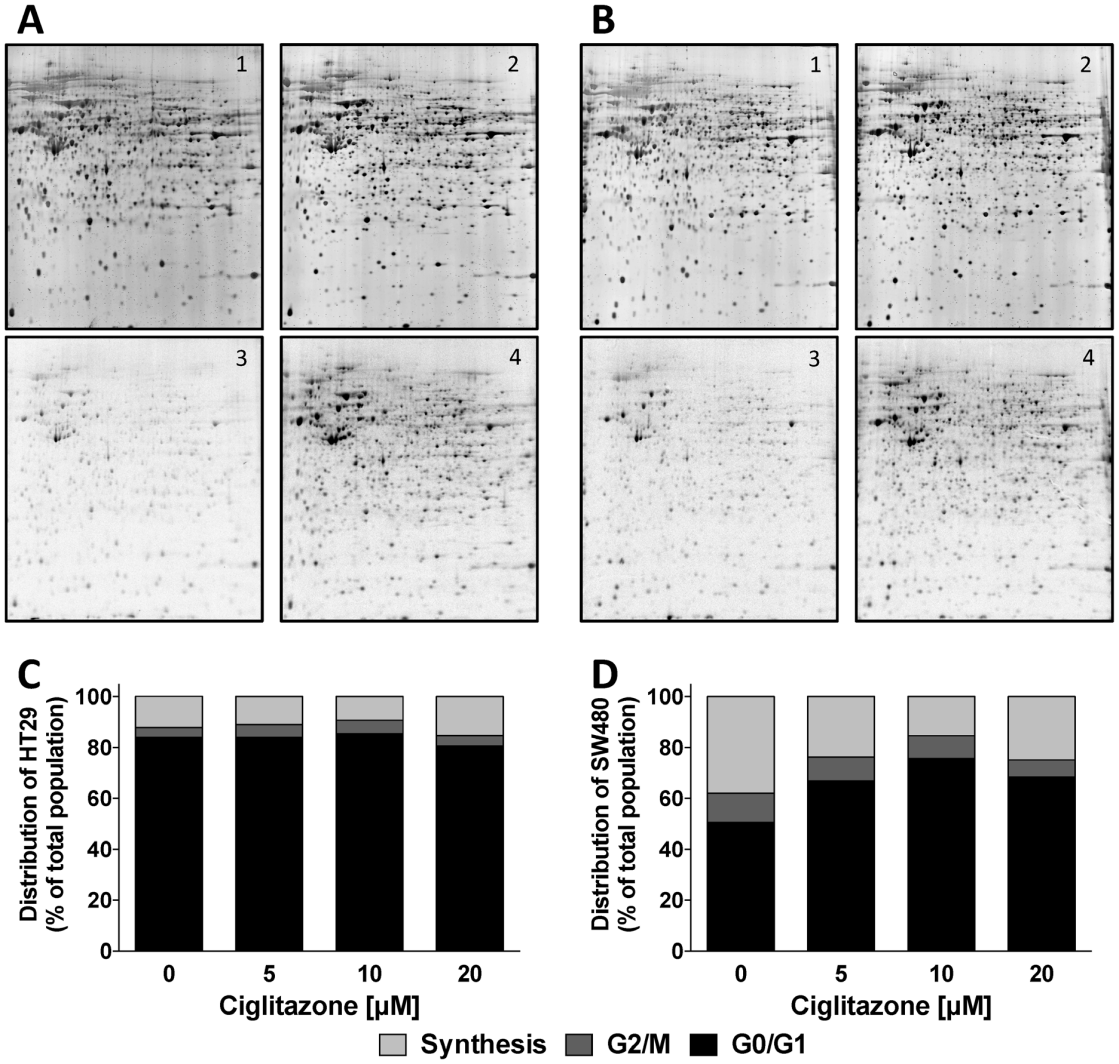
Cell responsiveness of colorectal adenocarcinoma cell lines after treatment with Ciglitazone. 2D-PAGE gels were performed in (A) HT29 and (B) SW480 cells after treatment with 5 µM Ciglitazone. (1) Fluorescence scans of untreated cells, (2) fluorescence scans of cells treated with Ciglitazone, (3) autoradiography scans of untreated cells, (4) autoradiography scans of cells treated with Ciglitazone. Cell cycle distribution of (C) HT29 and (D) SW480 cells treated with increasing concentrations of Ciglitazone.

Protein amounts determined by fluorescence detection were hardly affected upon treatment with CIG at 5 µM (Fig. 3A and 3B upper panel). Not a single protein was found up- or downregulated more than three-fold by CIG treatment in a significant manner. Since most proteins detected by fluorescence are relatively abundant, measurement of total protein amounts was not sensitive enough to assess the cells' responsiveness to CIG. In order to compare cell responsiveness to drug treatment in a more sensitive fashion we performed autoradiography scans [26], [27]. Cells treated with CIG at 5 µM responded with an upregulation of protein synthesis as compared to cells incubated with DMSO. In HT29 cells the synthesis was increased by 91±40% versus 74±26% in SW480 cells, indicating a slightly higher responsiveness of HT29 cells.

These results were supported by analysis of cell cycle distribution in HT29 and SW480 cells after exposure to CIG at 5 µM for 24 hours. Cell cycle distribution in isolated nuclei differed both at baseline and in response to treatment with CIG in both cell lines. In cultures of HT29 cells, only about 15% of the nuclei were in S-phase, while the G1 population was larger than 80% even in the control cultures (Fig. 3C). Cell cycle distribution did not change after CIG treatment. In contrast, 40% of nucei in SW480 cells were in the S-phase at baseline (Fig. 3D). After addition of CIG at 5 µM, nuclei accumulated in G1 in a concentration-dependent manner.

Chaperones were the proteins upregulated to the greatest extent after treatment with CIG in both cell lines (Table 2). The main function of chaperones is the folding of newly synthesized or misfolded proteins, enabling the cells to cope with stressors. Heat shock protein 105 kDa (HSP105) and endoplasmin were upregulated to a greater extent in SW480 than HT29 cells (Table 2). Miyazaki et al. reported that DNA vaccination of HSP105 resulted in tumor rejection of colorectal cancer in mice [57]. Furthermore, Liu et al. recently demonstrated the critical role of endoplasmin in the regulation of gut homeostasis in mice [58]. Loss of endoplasmin resulted in deregulation of Wnt signaling with deranged intestinal homeostasis, loss of crypt-villus structure, and reduced barrier function of the gut. Thus, the increased upregulation of chaperones and the already higher synthesis of chaperones in untreated SW480 cells pose a possible explanation for increased cell numbers in viability assays as well as a reduction in Δψ_m_ after treatment with CIG in SW480 cells.

**Table 2:** Protein regulation in SW480 and HT29 after treatment with 5 µM Ciglitazone as determined by 2D-PAGE autoradiography scans.

		SW480		HT29	
Protein	Acc.No.	Diff.	p-value	Diff.	p-value
*Heat-shock protein 105 kDa*	*Q92598*	***6,24***	***0,002***	*1,437*	*0,488*
*FK506-binding protein 4*	*Q02790*	***5,547***	***0,040***	*1.673*	*0,416*
*T-complex protein 1, theta subunit*	*P50990*	***3,806***	***0,004***	***4,142***	***0,017***
*Heat shock 70 kDa protein 1*	*P08107*	***3,649***	***0,037***	***3,641***	***0,016***
*Stress-70 protein*	*P38646*	***3,573***	***0,007***	***4,162***	***0,005***
*Endoplasmin*	*P14625*	***3,505***	***0,03***	*2,177*	*0,067*
Protein disulfide-isomerase A3	P30101	**3,133**	**0,01**	2,494	0,241
Tryptophanyl-tRNA synthetase	P23381	**3,074**	**0,034**	**4,214**	**0,017**
Transitional endoplasmic reticulum ATPase	P55072	**3,046**	**0,023**	1,964	0,056
*T-complex protein 1, beta subunit*	*P78371*	*1,644*	*0,048*	***3,104***	***0,019***

Proteins upregulated more than three-fold with statistical significance (p<0.05) are indicated by bold letters. Proteins involved in chaperoning functions are displayed in italic font.

The same treatment procedure with CIG at 5 µM was performed with both cell lines for analysis of the cytosolic protein fraction with a shotgun technique. We were able to identify about 1000 proteins in all four groups: 1010 proteins in untreated HT29 cells (S3 Table), 998 proteins in CIG-treated HT29 cells (S4 Table), 1011 proteins in untreated SW480 cells (S5 Table), and 1005 proteins in CIG-treated SW480 cells (S6 Table). 93% of proteins identified in 2D-experiments could also be verified using the shotgun approach. Taking into account the diverse setups of both experimental procedures the similar results imply high reliability of these data sets.

Drug treatments lead to dramatic changes in a cell's homoeostasis. Even when a substance has one target within the cell, multiple proteins are regulated through various signaling cascades. Thus, proteomics is an adequate method to observe regulatory mechanisms and approach the enigma of drug resistance from a broad angle. In the current study, we demonstrated that both baseline synthesis of chaperones and upregulation of proteins after treatment mediate the diverse responsiveness of HT29 and SW480 cells after stimulation with low-molar concentrations of TZDs. These results highlight the importance of knowing detailed protein patterns before attempting therapy of cancer.

## Supporting Information

S1 Figure Luciferase reporter assays.
(PDF)

S2 Figure Lactate dehydrogenase release into the medium of HT29 and SW480 cells after treatment with increasing concentrations of Ciglitazone for 24 hours.
(PDF)

S1 Table Protein list of shotgun analysis of HT29 and SW480 cells. Uniprot accession numbers, protein names and subcellular fractions are indicated. The listed numbers correspond to distinct peptides identified per protein in the indicated cell type. The robustness of identification is indicated by the number of positive Ids in relation to the number of shotgun analysis experiments. Furthermore, the exponentially modified Protein Abundance Index (emPAI) of each identification is indicated.
(XLS)

S2 Table Proteins detected by shotgun analysis in either HT29 or SW480 cells only. Proteins are grouped according to their known or presumed functions.
(XLS)

S3 Table Protein lists of shotgun analysis of untreated HT29 cells. Only proteins reproducibly identified with at least two distinct peptides are listed. Uniprot accession numbers, protein names, molecular weight and pI values of each identification are indicated.
(XLS)

S4 Table Protein lists of shotgun analysis of Ciglitazone-treated HT29 cells. Only proteins reproducibly identified with at least two distinct peptides are listed. Uniprot accession numbers, protein names, molecular weight and pI values of each identification are indicated.
(XLS)

S5 Table Protein lists of shotgun analysis of untreated SW480 cells. Only proteins reproducibly identified with at least two distinct peptides are listed. Uniprot accession numbers, protein names, molecular weight and pI values of each identification are indicated.
(XLS)

S6 Table Protein lists of shotgun analysis of Ciglitazone-treated SW480 cells. Only proteins reproducibly identified with at least two distinct peptides are listed. Uniprot accession numbers, protein names, molecular weight and pI values of each identification are indicated.
(XLS)
