# Supplementary figures and images for: Autonomous Inhibition of Apoptosis Correlates with Responsiveness of Colon Carcinoma Cell Lines to Ciglitazone
Source: PLoS One. 2014 Dec 11;9(12):e114158. doi: 10.1371/journal.pone.0114158 (PMC4263530; doi:10.1371/journal.pone.0114158)

# Supporting Information Figure 1

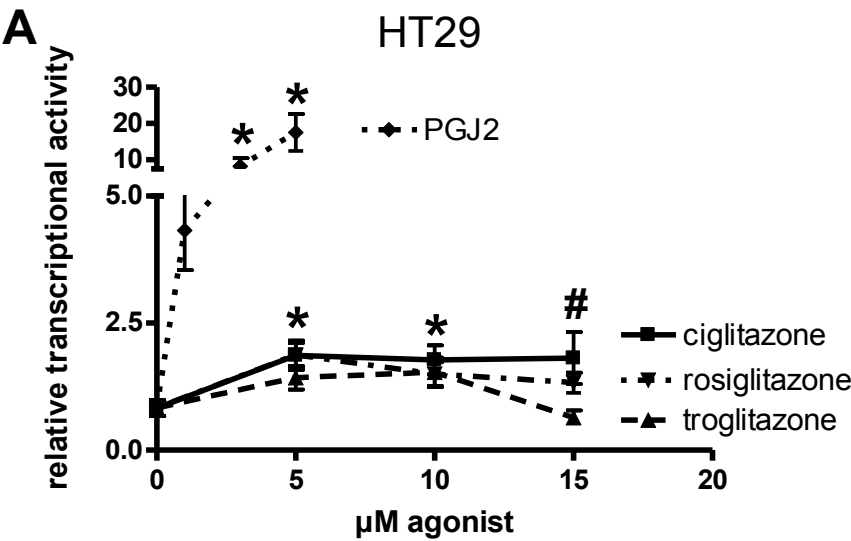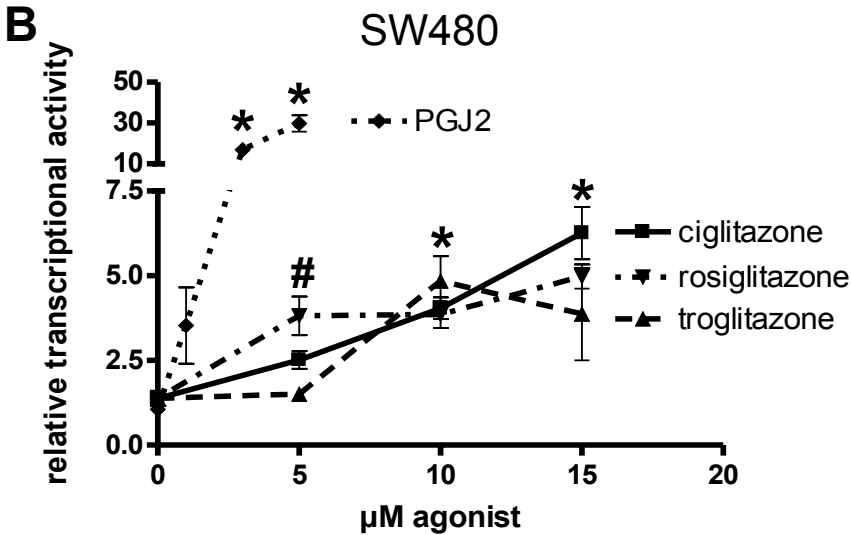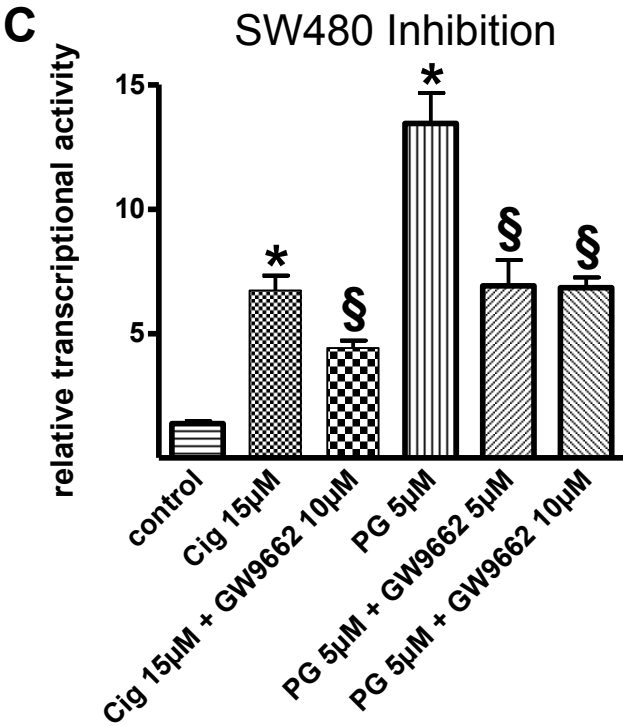

Supplement: S1 Figure — Luciferase reporter assays. (PDF) [file pone.0114158.s001.pdf]

# Supporting Information Figure 2

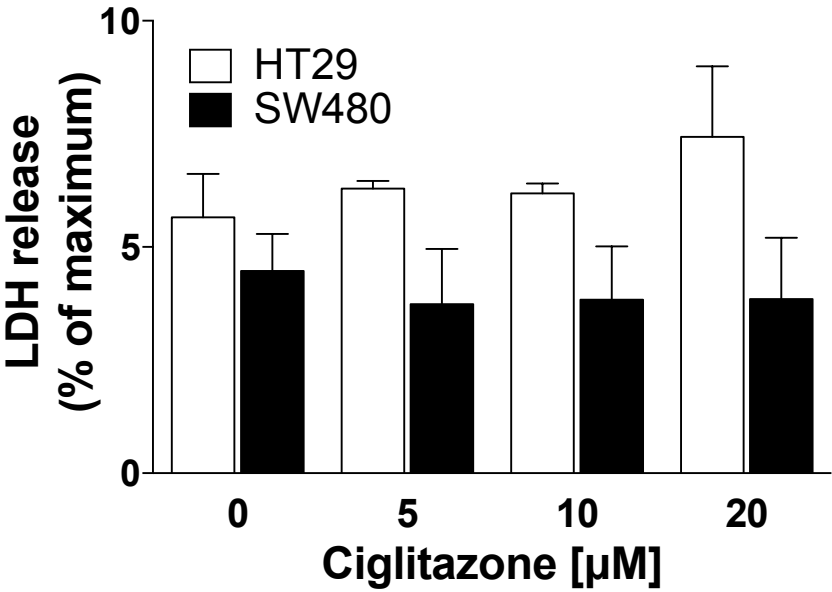

Supplement: S2 Figure — Lactate dehydrogenase release into the medium of HT29 and SW480 cells after treatment with increasing concentrations of Ciglitazone for 24 hours. (PDF) [file pone.0114158.s002.pdf]
